# Association Between Prostate Morphology and Intraoperative Blood Loss During Transurethral Resection of the Prostate: A Prospective Observational Study

**DOI:** 10.1155/aiu/6829187

**Published:** 2026-08-27

**Authors:** Yutong Lu, Guiling Wang, Jiangtao Bai, Haitao Jia, Enguang Yang, Xiangbin Kong, Qihui Zheng, Zhiping Wang

**Affiliations:** ^1^ Department of Urology, The Second Hospital of Lanzhou University, Lanzhou, China, lzu.edu.cn; ^2^ Gansu Province Clinical Research Center for Urinary System Diseases, Lanzhou, China; ^3^ Department of Anesthesiology, The Second Hospital of Lanzhou University, Lanzhou, China, lzu.edu.cn

**Keywords:** benign prostatic hyperplasia, blood loss, prostate morphology, transurethral prostatic resection

## Abstract

**Objective:**

To evaluate the association between prostate morphology and intraoperative blood loss during TURP.

**Materials and Methods:**

This prospective observational study included 1120 patients undergoing TURP. Intraoperative blood loss was quantitatively assessed using an endoscopic surgical monitoring system. The transverse and longitudinal diameters of the prostate were measured preoperatively by ultrasonography, and the W/L ratio was defined as the transverse‐to‐longitudinal diameter ratio. Univariable and multivariable linear regression analyses, nested regression models, and ROC analysis were used to evaluate the association between the W/L ratio and intraoperative blood loss.

**Results:**

The median intraoperative blood loss was 104 mL (IQR 36–214), and the median W/L ratio was 1.06 (IQR 1.02–1.18). Univariable analysis showed that the W/L ratio was negatively associated with intraoperative blood loss. In the multivariable analysis, after adjustment for age, BMI, prostate volume, operative time, *Q*_max_, 5‐ARI use, and surgeon experience category, the W/L ratio remained independently associated with intraoperative blood loss (*B* = – 45.957, 95% CI: −89.229 to −2.684, *p* = 0.037). Nested regression models showed that the addition of the W/L ratio increased the model *R* ^2^ from 0.444 to 0.446, indicating statistically significant but limited incremental information. Exploratory ROC analyses yielded AUCs of 0.816 and 0.791 for intraoperative blood loss thresholds of ≥ 300 mL and ≥ 400 mL.

**Conclusions:**

A lower prostate W/L ratio was independently associated with greater intraoperative blood loss during TURP. Although its effect size was smaller than those of operative time and prostate volume, the W/L ratio may serve as an adjunctive morphometric marker that is readily available from preoperative imaging.

**Trial Registration:** Chinese Registry of Clinical Trial: ChiCTR2400088743.

## 1. Introduction

Benign prostatic hyperplasia (BPH) is histologically present in approximately 50% of men aged 51–60 years, frequently manifesting as lower urinary tract symptoms (LUTSs) that substantially impair quality of life [1, 2]. While medical therapy remains first‐line for mild‐to‐moderate disease, transurethral resection of the prostate (TURP) is the established reference standard for moderate‐to‐severe symptomatic BPH refractory to pharmacotherapy or requiring immediate relief of bladder outlet obstruction [3, 4]. Nevertheless, intraoperative hemorrhage continues to represent a major source of perioperative morbidity, compromising surgical visualization, prolonging operative time, increasing transfusion requirements, and elevating the risk of adverse events [5, 6].

Previous studies have associated greater prostate volume, longer operative time, surgeon experience category, and anticoagulant or antiplatelet therapy with TURP‐related bleeding [7–9]. However, bleeding outcomes vary even among patients with similar prostate volumes and operative characteristics, suggesting that additional anatomical factors may be relevant. BPH exhibits pronounced morphological heterogeneity [10–12]. These architectural differences may theoretically modulate the extent of vascularized tissue exposed during resection, the complexity of hemostasis, and the accessibility of arterial feeders. Despite this mechanistic plausibility, prospective evidence regarding the association between objective morphological indices of BPH growth pattern and intraoperative blood loss remains limited.

To address this knowledge gap, the present large prospective observational study assessed whether prostate morphology, quantified using the width‐to‐length (W/L) ratio, was associated with intraoperative blood loss during TURP. The study hypothesis was that a lower W/L ratio, indicating a relatively elongated gland, would remain associated with greater blood loss after adjustment for relevant clinical and operative factors, including total prostate volume and operative duration. Prostate morphology was characterized using preoperative ultrasound measurements. Intraoperative blood loss was objectively quantified using a validated real‐time endoscopic surgical monitoring system (ESMS), which has shown reliable measurement performance in previous studies [13–17].

## 2. Patients and Methods

### 2.1. Study Design and Ethical Considerations

This prospective observational study enrolled consecutive patients undergoing TURP at Lanzhou University Second Hospital between June 2020 and November 2025. The study was conducted in accordance with the Declaration of Helsinki and approved by the Institutional Ethics Committee of Lanzhou University Second Hospital (No. 2020A‐275). Written informed consent was obtained from all participants before enrollment. The study was registered with the Chinese Clinical Trial Registry.

The study evaluated the association between prostate morphology and intraoperative blood loss during TURP. The preoperatively measured prostate W/L ratio was defined as the primary morphometric exposure, and intraoperative blood loss was defined as the primary outcome. Secondary intraoperative measures included irrigation fluid absorption, total irrigation volume, and operative time.

Owing to the exploratory observational nature of this study and the lack of reliable prior data quantifying the association between the W/L ratio and ESMS‐measured intraoperative blood loss during TURP, no formal a priori sample size calculation was performed. Consecutive eligible patients were enrolled during the study period, and the final sample size was determined by the number of patients with complete and valid clinical, imaging, and intraoperative data.

### 2.2. Study Population and Eligibility Criteria

Patients aged ≥ 50 years undergoing elective TURP for BPH were enrolled if they presented with a maximum urinary flow rate (*Q*_max_) ≤ 10 mL/s and an International Prostate Symptom Score (IPSS) ≥ 8, or a documented history of at least one episode of acute urinary retention. Additional inclusion criteria necessitated a confirmed diagnosis of BPH via clinical examination and auxiliary imaging, alongside symptoms refractory to standardized pharmacological therapies or recurrent urinary tract infections.

Preoperative exclusion criteria included severe cardiovascular, cerebrovascular, hepatic, or renal dysfunction precluding tolerance of surgery or anesthesia; severe coagulation disorders; inability to discontinue anticoagulant or antiplatelet therapy for at least 2 weeks before surgery; and concomitant bladder stones requiring simultaneous surgical treatment. At enrollment, previous use of 5‐alpha reductase inhibitors and antithrombotic agents was recorded.

Enrolled patients were excluded from the final analysis if TURP could not be completed because of urethral stricture. Patients were also excluded if prostatic capsular perforation, equipment or ESMS malfunction, substantial irrigation fluid spillage, incomplete hemostatic assessment, or missing key preoperative data made it impossible to evaluate operative time or intraoperative blood loss in a complete and reliable manner.

### 2.3. Data Acquisition and Surgical Protocol

Baseline demographic characteristics, preoperative clinical and laboratory data, imaging findings, and intraoperative variables were recorded prospectively. The longitudinal, transverse, and anteroposterior diameters of the prostate were measured by preoperative ultrasonography, and prostate volume was calculated from these measurements.

All TURP procedures were performed at Lanzhou University Second Hospital by urologists with more than 5 years of surgical experience. Intraoperative tranexamic acid use and surgeon experience category were recorded for each patient. Surgeon experience was categorized as 5–10 years or > 10 years. During surgery, the ESMS was connected to the fluid‐collection system through external suction tubing. Irrigation was performed with 0.9% saline, with the irrigation bags positioned 200 cm above the floor to maintain consistent hydrostatic pressure. All procedures followed the institutional TURP protocol. The operative procedure was not modified in response to ESMS monitoring unless a prespecified alert threshold was reached.

Given the specific focus of this study on the association between prostate morphology and intraoperative blood loss during TURP, prostatic vascular characteristics, including Doppler‐derived blood‐flow parameters and histological microvessel density, were not prospectively collected and were therefore not included in the analyses.

### 2.4. Statistical Analysis

The distribution of continuous variables was assessed using the Shapiro–Wilk test. Normally distributed variables are presented as mean ± standard deviation, non‐normally distributed variables as median (interquartile range), and categorical variables as number (percentage). Missing data were handled using complete‐case analysis. Univariable and multivariable linear regression analyses were performed to identify factors associated with intraoperative blood loss. The primary multivariable model included the W/L ratio, age, BMI, prostate volume, operative time, *Q*_max_, 5‐ARI use, and surgeon experience category.

Intraoperative tranexamic acid use was uncommon (21 patients, 1.9%) and was therefore not included in the primary multivariable model because of the risk of unstable coefficient estimates. Additional univariable and multivariable analyses excluding these patients are presented in the Supporting. In addition, tPSA was evaluated in the univariable analysis but was not included in the primary multivariable model because prostate volume was considered a more direct anatomical measure and tPSA may be influenced by both prostate volume and 5‐ARI therapy.

Multicollinearity was assessed using variance inflation factors. Nested models were constructed using hierarchical regression, and *R* ^2^, adjusted *R* ^2^, change in *R* ^2^, and F change were compared across model steps. An exploratory receiver operating characteristic (ROC) curve analysis was performed to assess the within‐cohort discrimination of the W/L ratio for higher intraoperative blood loss. All tests were two‐sided, and *p* < 0.05 was considered statistically significant. All analyses were performed using IBM SPSS Statistics Version 31.0.

## 3. Results

### 3.1. Participants

**FIGURE 1:**
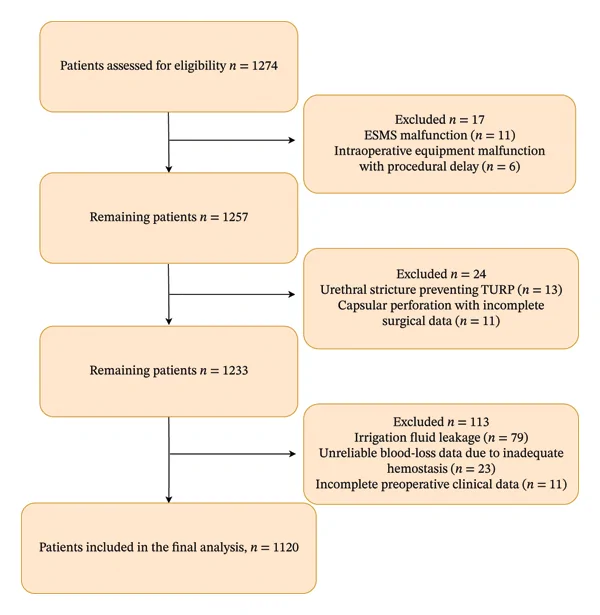
Study flow diagram.

Between June 2020 and November 2025, 1274 patients scheduled for TURP were assessed for eligibility (Figure 1). Of these, 154 were excluded, leaving 1120 patients in the final analysis. The most common reason for exclusion was incomplete or unreliable clinical or intraoperative data (*n* = 113), followed by surgery‐related factors (*n* = 24) and equipment failure (*n* = 17).

All patients presented with LUTSs secondary to benign prostatic obstruction and met the indications for TURP. The median prostate volume was 59 mL (IQR 39.4–84.25). Median operative time was 92 min (IQR 65–118), and the median volume of absorbed irrigation fluid was 1171 mL (IQR 557–1941). Median intraoperative blood loss was 104 mL (IQR 36–214). In the analysis of prostatic shape configurations, the median ratio of transverse to longitudinal diameter (W/L) was 1.06 (IQR 1.02–1.18). (see Table 1)

**TABLE 1:** The demographic and clinical characteristics of the included patients.

Variable	Data
Age, years, median (IQR)	71 (63,75)
BMI, kg/m^2^, median (IQR)	24.16 (20.43,25.21)
IPSS, score, median (IQR)	21 (16, 26)
*Q*_max_, mL/s, median (IQR)	7 (5,10)
Residual urine volume, mL, median (IQR)	13 (1121)
Positive preoperative urine culture, *n* (%)	271 (24.2)
Hypertension, *n* (%)	365 (32.59)
Cardiac disease, *n* (%)	271 (24.2)
Diabetes, *n* (%)	348 (31.07)
Other disease, *n* (%)	81 (7.23)
Prostate volume (PV), mL, median (IQR)	59 (39.4,84.25)
Prostate width, mm, median (IQR)	51 (47,60)
Prostate length, mm, median (IQR)	48 (37,56)
Prostate thickness, mm, median (IQR)	45 (38,52)
Prostate width to prostate length (W/L), median (IQR)	1.06 (1.02,1.18)
Prostate thickness to prostate length (T/L), median (IQR)	0.98 (0.89,1.09)
t‐PSA, ng/mL median (IQR)	4.35 (2,8.9)
f/t PSA ratio, median (IQR)	0.16 (0.11, 0.29)
Preoperative medical therapy, *n* (%)	
5‐ARI therapy, *n* (%)	884 (78.9)
No 5‐ARI therapy, *n* (%)	236 (21.07)
Intraoperative use of tranexamic acid, *n* (%)	21 (1.87)
Surgeon experience category, *n* (%)	
5–10 years	849 (75.8)
> 10 years	271 (24.2)
Operative time, min, median (IQR)	92 (65,118)
Total irrigation volume, mL, median (IQR)	26,140 (18364,33,254)
Irrigation fluid absorption, mL, median (IQR)	1171 (557,1941)
Intraoperative blood loss, mL, median (IQR)	104 (36,214)

*Note:* *Q*_max_: maximum urinary flow rate, f/t‐PSA: the ratio of free prostate‐specific antigen to total prostate‐specific antigen.

Abbreviations: 5‐ARI, 5‐alpha reductase inhibitor; BMI, body mass index; IPSS, International Prostate Symptom Score; t‐PSA, total prostate‐specific antigen.

### 3.2. Regression Analyses of Intraoperative Blood Loss

Univariable linear regression analysis showed that the W/L ratio, age, BMI, prostate volume, operative time, *Q*_max_, and history of 5‐ARI therapy were significantly associated with intraoperative blood loss, whereas no statistically significant association was observed for the surgeon experience category or total PSA (Table 2).

**TABLE 2:** Results of univariable and multivariable regression models.

Univariable regression analysis					
Intraoperative blood loss					
Variable	B	*β* value	*p* value	95% CI	*R* ^2^
W/L ratio	−27.451	−0.42	< 0.001	(−45.375, −18.53)	0.176
Age	2.856	0.131	< 0.001	(1.498, 4.213)	0.017
PV	2.454	0.507	< 0.001	(2.192, 2.715)	0.258
Time	2.601	0.62	< 0.001	(2.408, 2.794)	0.384
*Q*_max_	−8.654	−0.162	< 0.001	(−11.96, −5.347)	0.026
BMI	1.829	0.059	0.049	(0.008, 3.65)	0.003
Surgeon	−0.927	−0.002	0.939	(−24.87, 23.01)	0.001
5‐ARI	−44.763	−0.105	< 0.001	(−69.763, −19.763)	0.011
tPSA	−0.011	−0.011	0.721	(−0.07, 0.049)	0.001

**Multivariate regression analysis**					

**Intraoperative blood loss**					

Variable	b value	β value	*p* value	95% CI	VIF
W/L ratio	−45.957	−0.049	0.037	(−89.229, −2.684)	1.102
Age	1.131	0.052	0.023	(0.157, 2.106)	1.036
PV	1.217	0.245	< 0.001	(0.955, 1.479)	1.456
Time	1.994	0.476	< 0.001	(1.781, 2.208)	1.346
*Q*_max_	−0.985	−0.018	0.429	(−3.428, 1.459)	1.095
BMI	0.360	0.01	0.663	(−1.072, 1.684)	1.024
Surgeon	5.338	0.013	0.564	(−12.815, 23.491)	1.034
5‐ARI	−21.632	−0.051	0.026	(−40.664, −2.599)	1.034
*R* ^2^	0.446				

*Note:* Time: operative time, W/L ratio: the ratio of the transverse diameter to the longitudinal diameter of the prostate gland; *Q*_max_: maximum urinary flow rate; Surgeon: surgeon experience category.

Abbreviations; 5‐ARI, 5‐alpha reductase inhibitor; PV: prostate volume.

In the multivariable analysis, after adjustment for relevant covariates, the W/L ratio remained independently and negatively associated with intraoperative blood loss (*B* = −45.957, *β* = −0.049, 95% CI: −89.229 to −2.684, *p* = 0.037). Age, prostate volume, and operative time were positively associated with intraoperative blood loss, while history of 5‐ARI therapy was negatively associated with blood loss. Among the included variables, operative time (*β* = 0.476) and prostate volume (*β* = 0.245) showed relatively larger standardized effects. The *R* ^2^ of the model was 0.446, and all variance inflation factors were < 1.5.

In the sensitivity analysis excluding the 21 patients who received intraoperative tranexamic acid, the W/L ratio remained inversely associated with intraoperative blood loss in both univariable and multivariable analyses. In the multivariable model, the W/L ratio was associated with lower intraoperative blood loss after adjustment for age, BMI, prostate volume, operative time, *Q*_max_, 5‐ARI use, and surgeon experience category (*B* = −39.48, 95% CI: −73.163 to −5.770; *p* = 0.023; Table S2). In a supporting model including only prostate volume and the W/L ratio (Table S1), prostate volume was positively associated with intraoperative blood loss (*B* = 2.415, standardized *β* = 0.487, 95% CI 2.156 to 2.674; *p* < 0.001). The W/L ratio remained inversely associated with blood loss (*B* = −79.512, standardized *β* = −0.084, 95% CI: −128.790 to −30.239; *p* = 0.002). The model explained 26.8% of the variance, compared with 25.8% for prostate volume alone (Δ*R* ^2^ = 0.010).

The results of the nested regression models are shown in Table 3. The base model, which included age, BMI, operative time, prostate volume, history of 5‐ARI therapy, surgeon experience category, and *Q*_max_, explained 44.4% of the variance in intraoperative blood loss (*R* ^2^ = 0.444; adjusted *R* ^2^ = 0.440; *P* < 0.001). After further inclusion of the W/L ratio, the model *R* ^2^ increased from 0.444 to 0.446, and the adjusted *R* ^2^ increased to 0.442, corresponding to an absolute *R* ^2^ increase of 0.002 (F change = 4.342, *p* = 0.037). These findings indicate that the W/L ratio provided statistically significant but limited incremental information beyond the conventional clinical variables.

**TABLE 3:** Results of the nested linear regression models.

Nested regression analysis					
Intraoperative blood loss					
Model	Variables included	*R* ^2^	Adjusted *R* ^2^	Δ*R* ^2^	F change (*p* value)
Model 1	Conventional variables	0.444	0.440	0.444	126.597 (< 0.001)
Model 2	Model 1 + W/L ratio	0.446	0.442	0.002	4.342 (0.037)

*Note:* Model 1 included age, BMI, operative time, PV, 5‐ARI use, surgeon experience category, and *Q*_max_. Model 2 included all variables in Model 1 plus the W/L ratio. For the W/L ratio in Model 2, *b* = −45.957, *β* = −0.049, 95% CI (−89.229, −2.684), *p* = 0.037.

### 3.3. Exploratory ROC Analysis of the W/L Ratio for Higher Intraoperative Blood Loss

**FIGURE 2:**
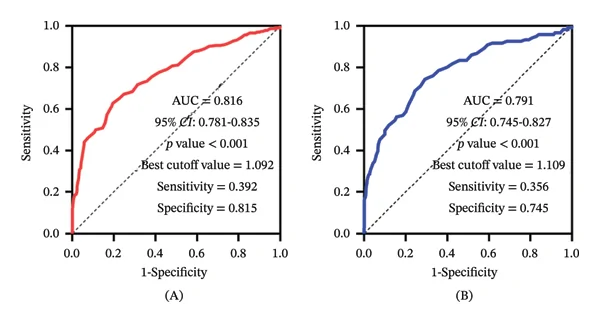
Exploratory discrimination of the prostate width‐to‐length ratio for higher intraoperative blood loss during TURP. (A) ROC curve for intraoperative blood loss ≥ 300 mL. (B) ROC curve for intraoperative blood loss ≥ 400 mL. The AUCs were 0.816 (95% CI 0.781–0.835) and 0.791 (95% CI 0.745–0.827), respectively.

Exploratory ROC curve analysis was performed to assess the discrimination of the W/L ratio for intraoperative blood loss thresholds of ≥ 300 mL and ≥ 400 mL. For blood loss ≥ 300 mL, the AUC was 0.816 (95% CI 0.781 to 0.835; *p* < 0.001), with a selected cutoff value of 1.092, a sensitivity of 0.392, and a specificity of 0.815. For blood loss ≥ 400 mL, the AUC was 0.791 (95% CI 0.745 to 0.827; *p* < 0.001), with a selected cutoff value of 1.109, a sensitivity of 0.356, and a specificity of 0.745. Although the AUCs indicated discriminatory ability within this cohort, the relatively low sensitivities limit the use of the W/L ratio as a standalone screening measure. (see Figure 2)

## 4. Discussion

Preoperative risk assessment may help anticipate intraoperative blood loss and guide perioperative management in patients undergoing TURP. In this prospective observational study, we examined whether prostate morphology, represented by the W/L ratio, was associated with intraoperative blood loss during TURP. After adjustment for age, BMI, prostate volume, operative time, *Q*_max_, 5‐ARI use, and surgeon experience category, a lower W/L ratio remained independently associated with greater intraoperative blood loss. Although operative time and prostate volume showed substantially larger standardized effects than the W/L ratio, this morphometric parameter provided limited incremental explanatory information. Therefore, the W/L ratio may serve as an easily obtainable preoperative adjunctive morphometric marker for estimating intraoperative blood loss.

Previous anatomical studies have shown that the prostate and bladder neck regions have a rich vascular supply, mainly derived from the urethral branches of the inferior vesical artery, which commonly enter the prostate at the 1–5 and 7–11o’clock positions around the bladder neck [10, 11]. Given this vascular anatomy, the more extensive and repeated manipulation required for a longitudinally elongated gland may increase the likelihood of disrupting vessels in this region. Such a gland may be more difficult to encompass within a single endoscopic field, necessitating repeated adjustments of the viewing angle, transitions between resection planes, and precise electrocoagulation. These additional endoscopic maneuvers may prolong operative time and complicate hemostatic control, thereby contributing to greater intraoperative blood loss. Another possible anatomical pathway involves middle lobe enlargement and intravesical prostatic protrusion (IPP). Longitudinal enlargement may coexist with middle lobe enlargement in some patients. When accompanied by IPP, protruding tissue may require a broader resection field and more extensive manipulation around the bladder neck [18–20]. This greater resection complexity could further contribute to intraoperative bleeding. Although this pathway was not directly evaluated, it may represent one of several anatomical explanations for the observed association and warrants further investigation.

The nested models further clarified the incremental contribution of the W/L ratio. Adding the W/L ratio to conventional clinical variables resulted in a small but statistically significant increase in explained variance. Similarly, its addition to a model containing prostate volume alone increased the *R* ^2^ by 0.01. Exploratory ROC analysis indicated some within‐cohort discrimination for higher intraoperative blood loss; however, the relatively low sensitivity and absence of external validation limit its use as a screening or clinical decision‐making tool. These findings should therefore be considered exploratory and require confirmation in independent cohorts.

Intraoperative blood loss during TURP may be influenced by perioperative medication exposure. Consistent with randomized evidence suggesting that short‐term finasteride pretreatment may reduce perioperative blood loss during TURP, preoperative 5‐ARI use was associated with lower intraoperative blood loss in this cohort [21, 22]. Tranexamic acid may also affect intraoperative bleeding [23]; however, only 21 patients (1.9%) received it, and this limited exposure was not included in the multivariable model because it could have produced unstable coefficient estimates. In additional analyses excluding these patients, the inverse association between the W/L ratio and intraoperative blood loss remained significant. Similarly, although anticoagulant or antiplatelet agents were discontinued at least 2 weeks before surgery, residual effects of previous exposure cannot be excluded. These medication‐related factors should therefore be considered when interpreting the findings.

Operator‐related and other unmeasured factors may also have influenced intraoperative blood loss [24]. Surgeon experience category was included in the multivariable model, but individual surgeon identity was not recorded. Therefore, operator‐level effects cannot be excluded despite the absence of a statistically significant association with experience category. In addition, prostatic vascular characteristics, including Doppler‐derived blood‐flow parameters and histological microvessel density, were not prospectively collected, leaving the possibility of residual confounding. Nevertheless, after adjustment for the measured covariates, the W/L ratio remained inversely associated with intraoperative blood loss. Its standardized effect was similar in magnitude to those of age and 5‐ARI therapy but smaller than those of prostate volume and operative time.

Existing studies of prostate morphology have focused primarily on the effects of middle lobe enlargement and IPP on voiding function and bladder outlet obstruction [25], whereas the association between overall prostate morphology and perioperative safety has not been systematically investigated. The present study extends this perspective by examining the association between overall prostate morphology and intraoperative blood loss during TURP. Nevertheless, the predictive value of the W/L ratio should be interpreted in conjunction with prostate volume, medication history, and other perioperative factors, rather than as an independent or standalone basis for risk assessment.

A methodological strength of the present study is the use of ESMS for real‐time quantitative assessment of intraoperative blood loss. Conventional visual estimation is inherently subjective, with measurement errors reported to reach 30%–50%. In contrast, ESMS determines the hemoglobin concentration in irrigation fluid using spectrophotometry, with a mean percentage error of approximately 5.2% [13, 17]. Compared with visual estimation, this method may reduce measurement bias related to the surgeon’s subjective judgment. However, ESMS depends on relatively complete collection of irrigation fluid; therefore, substantial irrigation fluid spillage or equipment failure may still compromise measurement validity.

Several limitations should be acknowledged. First, a formal a priori sample size calculation was not performed because reliable estimates of the expected effect size were not available from previous studies. Second, some patients were excluded because of incomplete data or unreliable measurements, and a complete comparison between included and excluded patients could not be performed. Therefore, selection bias cannot be completely excluded. Third, preoperative ultrasonography did not directly measure IPP, the extent of median lobe enlargement, or Doppler‐derived vascular parameters; thus, the relationships between the W/L ratio and these anatomical or vascular features could not be evaluated. Finally, this was a single‐center study, and the external applicability of the findings requires further validation in multicenter prospective studies.

## 5. Conclusion

A lower prostate W/L ratio was independently associated with greater intraoperative blood loss during TURP. Although its effect size was smaller than those of operative time and prostate volume, the W/L ratio may serve as an adjunctive morphometric marker that is readily available from preoperative imaging.

## Funding

This study was supported by the National Natural Science Foundation of China (No. 82060459), The Gansu Provincial Key Science and Technology Research Project (No. 24ZDFA002), The Gansu Provincial Science and Technology Plan (No. 25JRRA1032), the Cui Ying Science and Technology Innovation Plan of the Clinical Medicine School of Lanzhou University Second Hospital (No. CY2024‐YB‐A03), and The Gansu Provincial Science and Technology Commissioner Special Project (No. 25CXGA041).

## Conflicts of Interest

The authors declare no conflicts of interest.

## Supporting Information

Additional supporting information can be found online in the Supporting Information section.

## Supporting information


**Supporting Information** The following Supporting Material contains detailed files. Table S1 gives the results of three multiple linear regression models, and Table S2 shows univariate and multivariate linear regression analyses after excluding patients who received tranexamic acid during surgery.

## Data Availability

The data that support the findings of this study are available on request from the corresponding author. The data are not publicly available due to privacy or ethical restrictions.

## References

[1] Wei J. T. , Dauw C. A. , and Brodsky C. N. , Lower Urinary Tract Symptoms in Men: A Review, Journal of the American Medical Association. (2025) 334, no. 9, 10.1001/jama.2025.7045. 40658396

[2] Lerner L. B. , McVary K. T. , Barry M. J. et al., Management of Lower Urinary Tract Symptoms Attributed to Benign Prostatic Hyperplasia: Aua Guideline Part I-Initial Work-Up and Medical Management, Journal D’Urologie. (2021) 206, no. 4, 806–817, 10.1097/ju.0000000000002183. 34384237

[3] Schroeck F. R. , Hollingsworth J. M. , Kaufman S. R. , Hollenbeck B. K. , and Wei J. T. , Population Based Trends in the Surgical Treatment of Benign Prostatic Hyperplasia, Journal D’Urologie. (2012) 188, no. 5, 1837–1841, 10.1016/j.juro.2012.07.049. PMC4006217 22999698

[4] Cornu J. N. , Zantek P. , Burtt G. et al., Minimally Invasive Treatments for Benign Prostatic Obstruction: A Systematic Review and Network Meta-Analysis, European Urology. (2023) 83, no. 6, 534–547, 10.1016/j.eururo.2023.02.028. 36964042

[5] Aus G. , Bergdahl S. , Hugosson J. , and Norlén L. , Volume Determinations of the Whole Prostate and of Adenomas by Transrectal Ultrasound in Patients With Clinically Benign Prostatic Hyperplasia: Correlation of Resected Weight, Blood Loss and Duration of Operation, British Journal of Urology. (1994) 73, no. 6, 659–663, 10.1111/j.1464-410x.1994.tb07552.x. 7518318

[6] Zeng X. T. , Jin Y. H. , Liu T. Z. et al., Clinical Practice Guideline for Transurethral Plasmakinetic Resection of Prostate for Benign Prostatic Hyperplasia (2021 Edition), Military Medical Research. (2022) 9, no. 1, 10.1186/s40779-022-00371-6. PMC8974007 35361280

[7] ElMalik E. M. , Ibrahim A. I. , Gahli A. M. , Saad M. S. , and Bahar Y. M. , Risk Factors in Prostatectomy Bleeding: Preoperative Urinary Infection is the Only Reversible Factor, European Urology. (2000) 37, no. 2, 199–204, 10.1159/000020118. 10705199

[8] Hines L. , Doersch K. M. , Ninomiya M. , Jain R. , and Quarrier S. O. , Redefining Clinically Significant Hematuria After Holmium Enucleation of the Prostate, Journal of Endourology. (2023) 37, no. 11, 1216–1220, 10.1089/end.2023.0317. 37725558

[9] Descazeaud A. , Robert G. , Azzousi A. R. et al., Laser Treatment of Benign Prostatic Hyperplasia in Patients on Oral Anticoagulant Therapy: A Review, British Journal of Urology International. (2009) 103, no. 9, 1162–1165, 10.1111/j.1464-410x.2008.08284.x. 19154457

[10] Aaron L. , Franco O. E. , and Hayward S. W. , Review of Prostate Anatomy and Embryology and the Etiology of Benign Prostatic Hyperplasia, Urologic Clinics of North America. (2016) 43, no. 3, 279–288, 10.1016/j.ucl.2016.04.012. 27476121 PMC4968575

[11] Costa D. N. , Gupta A. C. , Shah R. B. , Cheney S. M. , and Yano M. , Anatomy of the Prostate and Periprostatic Region: A Contemporary Review, From the AJR Special Series on Critical Anatomy, American Journal of Roentgenology. (2025) 226. 10.2214/AJR.25.33454 40801496

[12] Gharbieh S. , Reeves F. , and Challacombe B. , The Prostatic Middle Lobe: Clinical Significance, Presentation and Management, Nature Reviews Urology. (2023) 20, no. 11, 645–653, 10.1038/s41585-023-00774-7. 37188789

[13] Bai J. , Jin Q. , Zheng Q. et al., In Vitro Evaluation of a Novel Automatic Intraoperative Blood Loss Monitor, Shock. (2023) 61, no. 5, 740–747, 10.1097/shk.0000000000002251. 38010043

[14] Jia H. , Zheng Q. , Zhang L. et al., Perioperative Hyperchloremia is Associated With Acute Kidney Injury in Elderly Patients Undergoing Bipolar Plasmakinetic Transurethral Resection of the Prostate: A Prospective Observational Study, World Journal of Urology. (2026) 44, no. 1, 10.1007/s00345-026-06503-0. 42189243

[15] Wang G. , Zhang L. , Jin Q. et al., Renal Pelvic Pressure Drives Fluid Absorption in mini-percutaneous Nephrolithotomy: A Quantitative Model for Absorption Rate, British Journal of Urology International. (2025) 136, no. 3, 529–536, 10.1111/bju.16812. 40484981

[16] Zhang L. , Yang E. , Jing S. et al., Risk Factors of High Fluid Absorption in Patients Treated With Mini-PCNL: A Single-Center Prospective Study, World Journal of Urology. (2024) 42, no. 1, 10.1007/s00345-024-04835-3. 38431764

[17] Zhang Y. , Fan N. , Zhang L. et al., Novel Strategy to Monitor Fluid Absorption and Blood Loss During Urological Endoscopic Surgery, Translational Andrology and Urology. (2020) 9, no. 3, 1192–1200, 10.21037/tau-19-780. 32676402 PMC7354347

[18] Gotoh D. , Torimoto K. , Tachibana A. et al., Efficacy and Safety of Thulium Laser Vaporization of the Prostate: A Transurethral Procedure for Benign Prostatic Hyperplasia, Luts. (2025) 17, no. 3, 10.1111/luts.70012. 40230044

[19] Bulbul E. , Oztekin O. , and Yavuz Ilki F. , Intravesical Prostatic Protrusion Volume: Effect on Lower Urinary Tract Symptoms and Urinary Flow in Patients With Benign Prostatic Enlargement, World Journal of Urology. (2025) 43, no. 1, 10.1007/s00345-025-05986-7. 41044460

[20] Liu Q. , Zhu Y. , Liu J. , Qi J. , and Kang J. , Ultrasound Image Features of Intravesical Prostatic Protrusion Indicated Failure of Medication Therapy of Finasteride and Doxazosin in Patients With Benign Prostatic Hyperplasia (LUTS/BPH), International Urology and Nephrology. (2016) 49, no. 3, 399–404, 10.1007/s11255-016-1478-6. 27987130

[21] Hehir C. M. , Calpin G. G. , Cullivan O. , Daly G. R. , Dowling G. P. , and Davis N. F. , The Role of 5-Alpha Reductase Inhibitors in Transurethral Resection of the Prostate: A Meta-Analysis of Randomised Controlled Trials, British Journal of Urology International. (2026) 137, no. 3, 430–443, 10.1111/bju.70117. PMC12907782 41502115

[22] Dutt U. K. , Kumar S. , Dorairajan L. N. , Badhe B. A. , Manikandan R. , and Singh S. , Effect of Preoperative Finasteride on Perioperative Blood Loss During Transurethral Resection of the Prostate and on Microvessel Density in Patients With Benign Prostatic Hyperplasia: An Open Label Randomized Controlled Trial, Urology Annals. (2021) 13, no. 3, 10.4103/ua.ua_35_20. PMC8343290 34421251

[23] Tikkinen K. A. O. , Marcucci M. , Halme A. L. E. et al., Safety and Efficacy of Tranexamic Acid in Urologic Surgery: Results From the International, Randomized, Placebo-Controlled POISE-3 Trial, European Urology. (2026) 0302-2838, no. 26. 10.1016/j.eururo.2026.03.019 42020258

[24] Riedinger C. B. , Fantus R. J. , Matulewicz R. S. , Werntz R. P. , Rodriguez J. F. , and Smith N. D. , The Impact of Surgical Duration on Complications After Transurethral Resection of the Prostate: An Analysis of NSQIP Data, Prostate Cancer and Prostatic Diseases. (2018) 22, no. 2, 303–308, 10.1038/s41391-018-0104-3. 30385836

[25] Chia S. J. , Heng C. T. , Chan S. P. , and Foo K. T. , Correlation of Intravesical Prostatic Protrusion With Bladder Outlet Obstruction, British Journal of Urology International. (2003) 91, no. 4, 371–374, 10.1046/j.1464-410x.2003.04088.x. 12603417

