# Supplementary material for: Association Between Prostate Morphology and Intraoperative Blood Loss During Transurethral Resection of the Prostate: A Prospective Observational Study
Source: Adv Urol. 2026 Aug 27;2026:6829187. doi: 10.1155/aiu/6829187 (PMC13522824; doi:10.1155/aiu/6829187)
Supplement: Supplementary file 1 [file AIU-2026-6829187-s001.zip › Supplement Table S1.docx]

Table S1 Results of three supplementary multivariable linear regression models

| Supplementary Multivariable Regression Analysis | | | | | |
| --- | --- | --- | --- | --- | --- |
|  | Intraoperative blood loss | | | | |
| Variable | B | β value | P value | 95% CI | VIF |
| Model 1: W/L ratio and PV (R² = 0.268) | | | | | |
| W/L ratio | -79.512 | -0.084 | 0.002 | (-128.790, -30.239) | 1.085 |
| PV | 2.415 | 0.487 | <0.001 | (2.156, 2.674) | 1.085 |
| Model 2: W/L ratio and operative time (R² = 0.393) | | | | | |
| W/L ratio | -92.514 | -0.098 | <0.001 | (-136.500, -48.531) | 1.043 |
| Time | 2.517 | 0.600 | <0.001 | (2.321, 2.713) | 1.043 |
| Model 3: W/L ratio, PV, and operative time (R² = 0.442) | | | | | |
| W/L ratio | -47.086 | -0.050 | 0.033 | (-90.255, -3.916) | 1.043 |
| PV | 1.283 | 0.259 | <0.001 | (1.028, 1.539) | 1.384 |
| Time | 2.021 | 0.482 | <0.001 | (1.809, 2.234) | 1.331 |

Dependent variable: intraoperative blood loss. *b*: unstandardized regression coefficient; *β*: standardized regression coefficient; CI: confidence interval; VIF: variance inflation factor; PV: prostate volume; Time: operative time; W/L ratio: the ratio of the transverse diameter to the longitudinal diameter of the prostate gland.
