# Supplementary material for: Association Between Prostate Morphology and Intraoperative Blood Loss During Transurethral Resection of the Prostate: A Prospective Observational Study
Source: Adv Urol. 2026 Aug 27;2026:6829187. doi: 10.1155/aiu/6829187 (PMC13522824; doi:10.1155/aiu/6829187)
Supplement: Supplementary file 1 [file AIU-2026-6829187-s001.zip › Supplement Table S2.docx]

| Univariable Regression Analysis | | | | | |
| --- | --- | --- | --- | --- | --- |
| Intraoperative blood loss | | | | | |
| Variable | B | β | P value | 95% CI | R² |
| W/L ratio | −30.32 | −0.392 | <0.001 | (-41.36, -19.27) | 0.154 |
| Age (years) | 3.065 | 0.14 | <0.001 | (1.784, 4.345) | 0.02 |
| BMI (kg/m²) | 1.927 | 0.063 | 0.038 | (0.103, 3.752) | 0.004 |
| PV (mL) | 2.554 | 0.513 | <0.001 | (2.300, 2.807) | 0.263 |
| Time (min) | 2.705 | 0.625 | <0.001 | (2.504, 2.905) | 0.39 |
| Qmax (mL/s) | −7.638 | −0.144 | <0.001 | (−10.757, −4.518) | 0.021 |
| 5-ARI | −41.106 | −0.096 | 0.002 | (−66.467, −15.745) | 0.009 |
| Surgeon | -2.213 | -0.005 | 0.864 | (−27.6, 23.2) | 0.001 |
| tPSA (ng/mL) | −0.011 | −0.011 | 0.726 | (−0.070, 0.049) | 0.001 |
| Multivariable Regression Analysis | | | | | |
| Intraoperative blood loss | | | | | |
| Variable | B | β | P value | 95% CI | VIF |
| W/L ratio | −39.48 | −0.41 | 0.023 | (−73.163, -5.770) | 1.109 |
| Age (years) | 1.129 | 0.052 | 0.024 | (0.151, 2.107) | 1.037 |
| BMI (kg/m²) | 0.308 | 0.01 | 0.66 | (−1.067, 1.683) | 1.025 |
| PV (mL) | 1.232 | 0.247 | <0.001 | (0.968, 1.497) | 1.45 |
| Time (min) | 2.086 | 0.482 | <0.001 | (1.865, 2.307) | 1.346 |
| Qmax (mL/s) | −0.917 | −0.017 | 0.462 | (−3.365, 1.531) | 1.091 |
| 5-ARI | −22.381 | −0.052 | 0.022 | (−41.548, −3.214) | 1.027 |
| Surgeon | 7.107 | 0.017 | 0.444 | (−11.109, 25.323) | 1.032 |
| R² | 0.452 | | | | |

Table S2. Univariable and multivariable linear regression analyses after excluding patients who received intraoperative tranexamic acid
